# MIRUS™, a new system for sedation with halogenates in the ICU: a preliminary study of feasibility in postsurgical patients

**DOI:** 10.1186/cc14572

**Published:** 2015-03-16

**Authors:** P Mancinelli, S Romagnoli, C Chelazzi, G Zagli, E Bonicolini, A Belardinelli, AR De Gaudio

**Affiliations:** 1 Azienda Ospedaliero-Universitaria Careggi, Florence, Italy

## Introduction

Sedation is standard practice in the ICU [1]. The aim of this study was to investigate the efficiency and safety of the MIRUS™ system (Pall Medical), a new device for sedation in ICU patients [2]. The system delivers volatile anesthetics in mechanically ventilated patients. An open reservoir scavenger and a dedicated gas filter avoid residual volatile anesthetic halogenate escaping into the room air.

## Methods

Ten mechanically ventilated patients electively admitted for ICU postoperative monitoring were sedated with sevoflurane delivered with the MIRUS™ system. Two patients were excluded from the analysis because inclusion criteria had been lost during the study period. Analgesia was obtained with morphine sulfate: bolus 0.1 mg/ kg i.v. at the end of surgery and 0.2 to 0.4 mg/kg/24 hours. The primary endpoint was to achieve predefinite levels of sedation (Riker scale 4). Secondary endpoints were the assessment of hemodynamic stability (MAP and HR), blood lactates, any type of side effects, and sevoflurane consumption. Data were collected at the following times: admission to the ICU (T1), 1 hour after initiation of sedation (T2), and 1 hour after sedation withdrawal (T3). Results were expressed as median (IQR) or mean (SD), where appropriate.

## Results

The local ethical board approved the protocol. Median duration of sedation was 4 (5.5 to 2) hours. Predefinite levels of sedation were achieved in all patients with a median MAC of sevoflurane of 0.5 (0.5 to 0.3)% and with a median gas consumption of 9.9 (14.3 to 5.3) ml/hour. MAP and HR values at T1 were 86.5 (97 to 80.8) mmHg and 81.5 (103.8 to 65) bpm, respectively; at T2, 74.5 (89 to 69.5) mmHg and 74 (82 to 58.3) bpm, respectively; and at T3 92.5 (101 to 76.8) mmHg and 74 (88.5 to 66.3) bpm, respectively. Lactates were always normal. Mechanical ventilation was interrupted 5.4 (3.1) minutes after withdrawal of sevoflurane and respiratory parameters always were within normal values. Finally, no side effects were registered at any phase of the study.

## Conclusion

This pilot study shows that MIRUS™ is effective and safe in delivering sevoflurane for sedation at a predefinite target level in postsurgical patients, without side effects. Further data with a larger number of patients and for a longer duration of sedation are required to confirm these positive, preliminary observations.
